# Breakdown of Universal Scaling for Nanometer-Sized Bubbles in Graphene

**DOI:** 10.1021/acs.nanolett.1c02470

**Published:** 2021-09-14

**Authors:** Renan Villarreal, Pin-Cheng Lin, Fahim Faraji, Nasim Hassani, Harsh Bana, Zviadi Zarkua, Maya N. Nair, Hung-Chieh Tsai, Manuel Auge, Felix Junge, Hans C. Hofsaess, Stefan De Gendt, Steven De Feyter, Steven Brems, E. Harriet Åhlgren, Erik C. Neyts, Lucian Covaci, François M. Peeters, Mehdi Neek-Amal, Lino M. C. Pereira

**Affiliations:** † Quantum Solid State Physics, KU Leuven, 3001 Leuven, Belgium; ‡ Research group PLASMANT, Department of Chemistry, Universiteit Antwerpen (UIA), 2610 Antwerpen, Belgium; ¶ Departement Natuurkunde, Universiteit Antwerpen (UIA), 2610 Antwerpen, Belgium; § Department of Physics, Shahid Rajaee Teacher Training University, 16875-163 Lavizan, Tehran, Iran; ∥ CUNY Advanced Science Research Center, 85 St. Nicholas Terrace, New York, New York 10031, United States; ⊥ imec vzw (Interuniversitair Micro-Electronica Centrum), 3001 Leuven, Belgium; # Department of Chemistry, Division of Molecular Design and Synthesis, KU Leuven, 3001 Leuven, Belgium; @ II.Institute of Physics, University of Göttingen, 37077 Göttingen, Germany; Δ Department of Chemistry, Division of Molecular Imaging and Photonics, KU Leuven, 3001 Leuven, Belgium; ∇ Faculty of Physics, University of Vienna, 1090 Vienna, Austria

**Keywords:** graphene, nanobubbles, aspect ratio, scanning tunneling microscopy

## Abstract

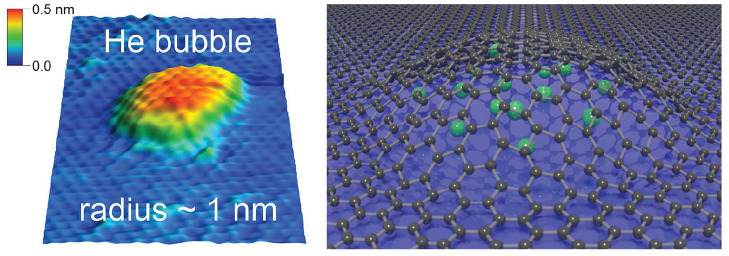

We report the formation of nanobubbles on graphene with a radius of the order of 1 nm, using ultralow energy implantation of noble gas ions (He, Ne, Ar) into graphene grown on a Pt(111) surface. We show that the universal scaling of the aspect ratio, which has previously been established for larger bubbles, breaks down when the bubble radius approaches 1 nm, resulting in much larger aspect ratios. Moreover, we observe that the bubble stability and aspect ratio depend on the substrate onto which the graphene is grown (bubbles are stable for Pt but not for Cu) and trapped element. We interpret these dependencies in terms of the atomic compressibility of the noble gas as well as of the adhesion energies between graphene, the substrate, and trapped atoms.

Owing to its unrivaled elasticity and strength,^1,2^ graphene is able to hold matter at extreme pressures in the form of bubbles with dimensions down to the nanometer scale.^3−6^ These bubbles offer new opportunities to explore chemistry and physics under the extreme conditions that both graphene and the trapped matter are subject to, for example, strain-induced pseudomagnetic fields in graphene^7−9^ and high-pressure chemical reactions.^10,11^ Similar nanobubbles in other 2D materials such as MoS_2_ and h-BN are also being investigated as single-photon emitters for quantum communication.^12,13^

While previous research has mostly dealt with bubbles with a radius of few nm and larger, the subnanometer regime remains largely unexplored. Here, we report the formation of graphene nanobubbles with a radius down to below 1 nm, filled with He, Ne, and Ar. Delving into the physical mechanisms that determine the stability and shape of these subnanometer bubbles reveals that they constitute a fundamentally different regime, exhibiting an extreme aspect ratio, tensile strain, and pressure. The unique properties of this subnanometer regime open an unexplored ground for applications of nanobubbles in 2D materials.

The properties of graphene bubbles with a radius of a few nm and larger are relatively well understood on the basis of elasticity theory as well as graphene’s elastic properties and its van der Waals (vdW) attraction to the substrate.^5^ In this regime, bubbles have been observed on various substrates (e.g., Ir, Pt, h-BN, SiO_2_) with a variety of trapped substances (e.g., water, noble gases, hydrocarbons),^3,5,6,14,15^ which do not appear to significantly affect the bubble stability.^5,6^ The substrate and trapped substance do affect key properties, such as shape (in particular the aspect ratio) and the pressure inside the bubble.^5,6^ This dependence is largely determined by the balance of the adhesion energies: between graphene and the substrate (γ_GS_), between the substrate and the trapped substance (γ_Sb_), and between the graphene and the trapped substance (γ_Gb_).^5^ A particularly striking feature demonstrated for nanobubbles in the few nm regime and larger is that the aspect ratio exhibits universal scaling
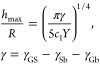
1where *h*_max_ is the bubble maximum height, *R* is the bubble radius at the base, *c*_1_ is a constant (0.7), and *Y* is the Young modulus.^5^ Here, by combining scanning tunneling microscopy (STM) measurements with molecular dynamics (MD) simulations and density functional theory (DFT) calculations, we show that this universal scaling breaks down at small *R* (near 1 nm and below). We also observe that the bubble stability is strongly dependent on the substrate. We interpret these dependencies in terms of the role of the atomic compressibility of the noble gases as well as of the adhesion energies (γ_GS_, γ_Sb_, and γ_Gb_). Moreover, these nanobubbles are found to induce high levels of strain (of the order of 10%) on the overlaying graphene and are predicted by our MD simulations to hold the noble gas atoms under extreme pressures (exceeding 30 GPa).

## Experimental Details and Basic Characterization

**Figure 1:**
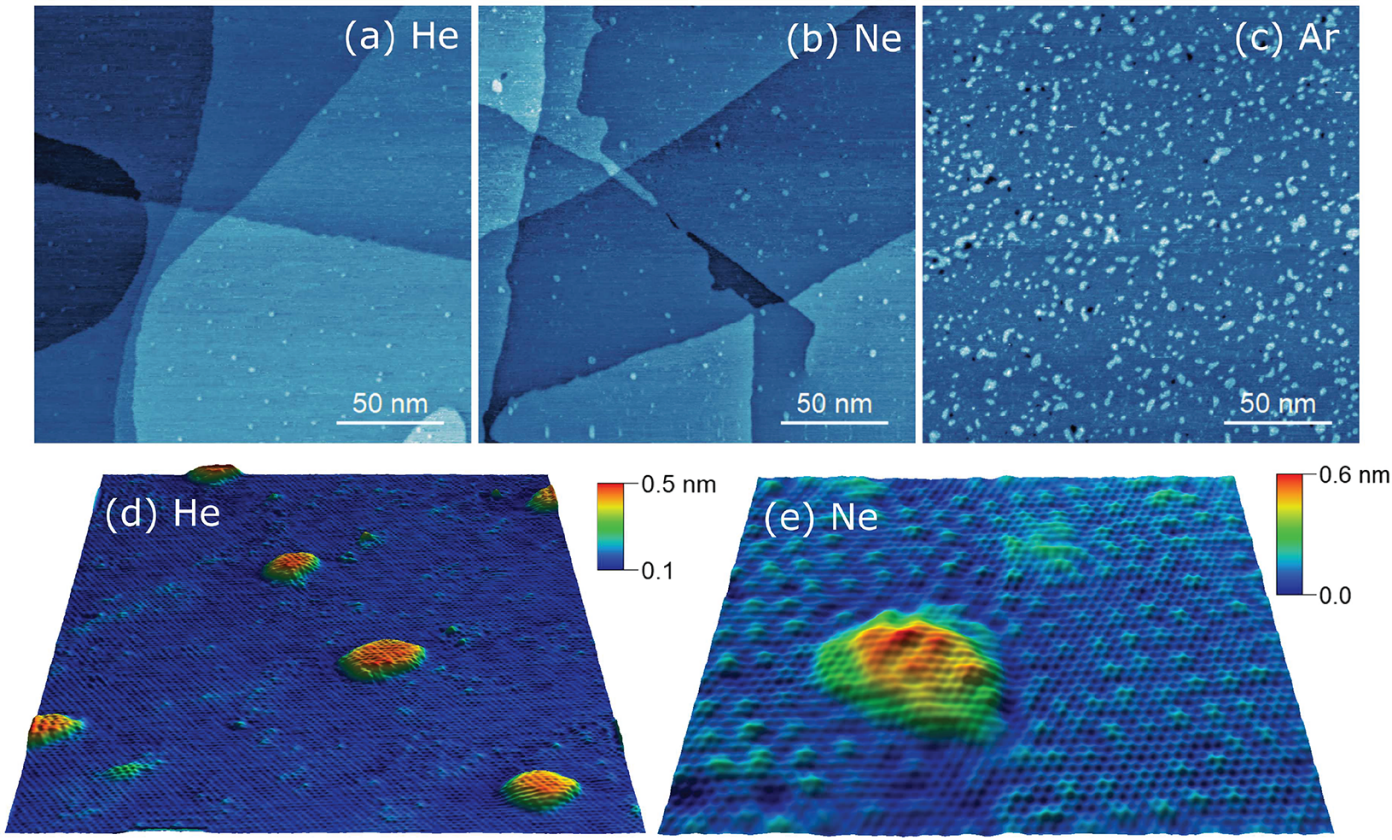
STM micrographs showing (a) He, (b) Ne, and (c) Ar bubbles in graphene/Pt(111). (d,e) STM micrographs (20 × 20 and 10 × 10 nm^2^, respectively), with atomic resolution, showing a continuous graphene atomic lattice, in particular, over the bubbles.

Our samples consist of epitaxial graphene grown by chemical vapor deposition (CVD) on epitaxial Pt(111) and Cu(111) thin films grown on sapphire(0001) substrates.^16,17^ Nanobubbles are formed by implanting noble gas ions (He, Ne, and Ar), with a kinetic energy of 25 eV, with perpendicular incidence with respect to the surface. Bubbles were found to only form for graphene on Pt(111) (Figure 1), not for graphene on Cu(111) (Figure S1). In the following, we will focus on Pt(111) and return to Cu(111) further below when discussing how the bubble stability depends on the substrate. Ion implantation has been previously used to form graphene nanobubbles of noble gases.^3,14,15^ In contrast to the previous studies, where ion beams with energies of 500 eV and higher were used, our approach is based on ultralow energy (ULE) ion implantation. Such low energies are crucial to minimize irradiation-induced damage. Based on our MD simulations (Figure S2), we selected 25 eV (surface normal incidence) as sufficiently high for a significant fraction of the ions to be transmitted through the graphene layer but sufficiently low to minimize carbon atom displacements (i.e., formation of vacancies and related point defects). While ULE ion implantation has been previously used for doping of graphene (e.g., with B and N^18−20^) where vacancies are required (which allows for substitutional incorporation of the dopant atoms), such defects must be avoided in the context of the present work so that the intrinsic elastic properties of graphene are maintained. The graphene bubbles observed in our samples are identified as nanometer-scale protrusions on the surface of graphene (grown on Pt(111), implanted with the noble gases) as shown in the STM topographies in Figure 1. The fact that the graphene lattice can be resolved even over these protrusions confirms that the implanted noble gases are intercalated (Figure 1d,e), that is, the protrusions are not due to matter deposited on top of graphene. The fraction of surface that is covered by bubbles (for the same implanted fluence) was found to vary between implanted noble gas elements (Figure 1a–c). This dependence is likely due to the different transmission and backscattering probabilities for the different elements (cf. Supporting Information). The high structural order of the irradiated surfaces is supported by our atomic-resolution STM measurements on the as-implanted surfaces (Figure 1d,e) and by the integrity of the moiré superstructure in most of the surface with only minor disorder (Figure 1e). This minor disorder is due to defects introduced during the implantation process, and it can be seen in the STM topographies as point-like features (protrusions and depressions) perturbing the periodicity of the atomic lattice (Figure 1d,e) and of the moiré superstructure (Figure 1e) (cf. Supporting Information). Indeed, Raman spectroscopy measurements show some degree of disorder (Figure S6). Since the selected implantation energy is below the threshold for vacancy formation (Figure S2), this disorder is likely associated with the breaking of C–C bonds without the production of C vacancies and likely resulting in locally enhanced interaction of the Pt atoms at the interface, leading to the subtle defect features observed by STM.

## Breakdown of Universal Scaling at Low Radius

**Figure 2:**
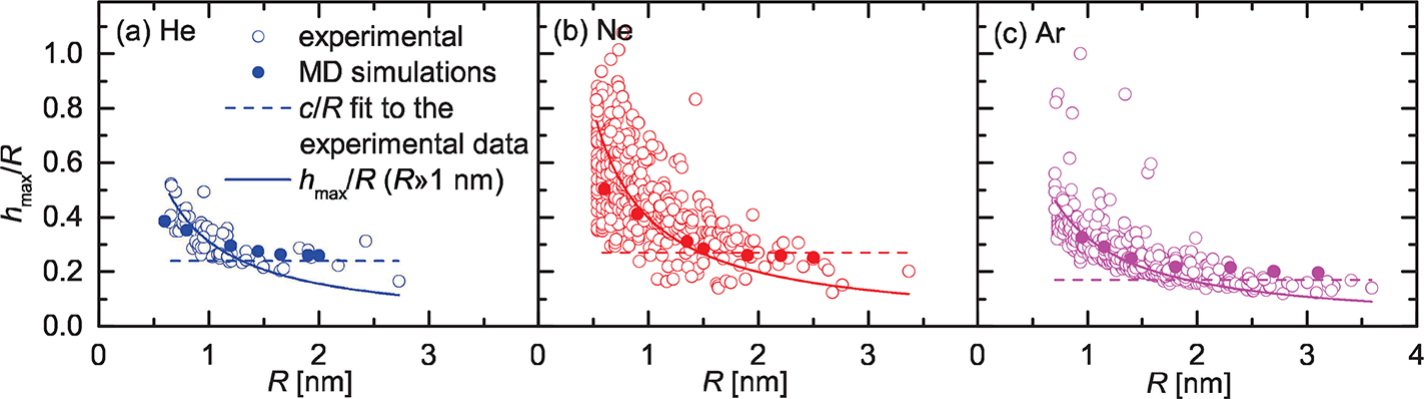
*h*_max_/*R* as a function of *R* obtained from STM micrographs such as those shown in Figure 1 (empty circles) and from MD simulations (filled circles), for (a) He, (b) Ne, and (c) Ar bubbles in graphene/Pt(111). Each experimental data point corresponds to one bubble. The solid line is a fit with the function (*h*_max_/*R* = *c*/*R*). The dotted line corresponds to the value of .

The radius and aspect ratio of each bubble, for the different elements (He, Ne, and Ar), are plotted in Figure 2. A clear trend is observed for all three gases. For larger *R* values (>1 nm), the aspect ratio tends to converge to a constant value of about 0.2, which is in agreement with the universal scaling previously observed for bubbles with a radius of few nm and larger.^5^ However, as *R* approaches the subnanometer regime, the universal scaling breaks down, showing an increase in the aspect ratio and approaching 1 for Ne bubbles. From the experimental data in Figure 2, we calculated, for each gas (He, Ne, Ar), an average value for *h*_max_^0^ (from the 10% smallest bubbles) and an average value for  (from the 10% largest bubbles). These values are compiled in Table 1.

**Table 1:** Aspect Ratio and Related Parameters^a^

element	*h*_max_^0^ [Å]	2*r*_vdW_ [Å]	β [au]	⟨Δ*z*⟩ [Å]	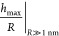	γ [eV·Å^–2^]
He	2.9(±0.5)	2.86	–0.152	0.31	0.24(±0.05)	0.08
Ne	3.5(±0.8)	3.16	–0.266	0.49	0.27(±0.07)	0.13
Ar	3.1(±0.6)	3.88	0.081	0.29	0.17(±0.03)	0.02

a *h*_max_^0^ and  are obtained from the data in Figure 2. *h*_max_^0^ is the average of *h*_max_ taken over the 10% smallest bubbles.  is the average  taken over the 10% largest bubbles. The values inside the brackets are the standard deviation associated with the respective averages. ⟨Δ*z*⟩ is the average *z*-motion amplitude obtained from the MD simulations, for the smallest bubbles (radius of ∼6 Å for He and Ne and ∼9 Å for Ar). γ is calculated using eq 1 with  given by . 2*r*_vdW_ is the vdW diameter (from ref (21)), and β is the atomic compressibility (from ref (22)).

Bending rigidity (neglected in the derivation of eq 1) becomes more important as the bubble dimensions decrease down to <1 nm.^5^ However, as described in the Supporting Information, the effect is still negligible for the bubbles described here and is in fact in the opposite direction (decreases the aspect ratio). The observed breakdown of the universal scaling must therefore originate from a different mechanism, namely the existence of a minimum value for *h*_max_ (*h*_max_^0^), corresponding to one atomic layer of the trapped gas atoms. As *R* approaches this regime, *h*_max_ becomes a constant value (*h*_max_^0^), and consequently, *h*_max_/*R* transits into a ∼1/*R* dependence. This is illustrated in Figure 2c by the fit to the experimental *h*_max_/*R* data with the function (*h*_max_/*R* = *c*/*R*), where *c* (around 3 to 4 Å) is comparable to *h*_max_^0^. This ∼1/*R* fit crosses the value corresponding to  (dotted line) around 1–2 nm, above which the universal scaling regime is valid and *h*_max_/*R* becomes constant, given by eq 1.

**Figure 3:**
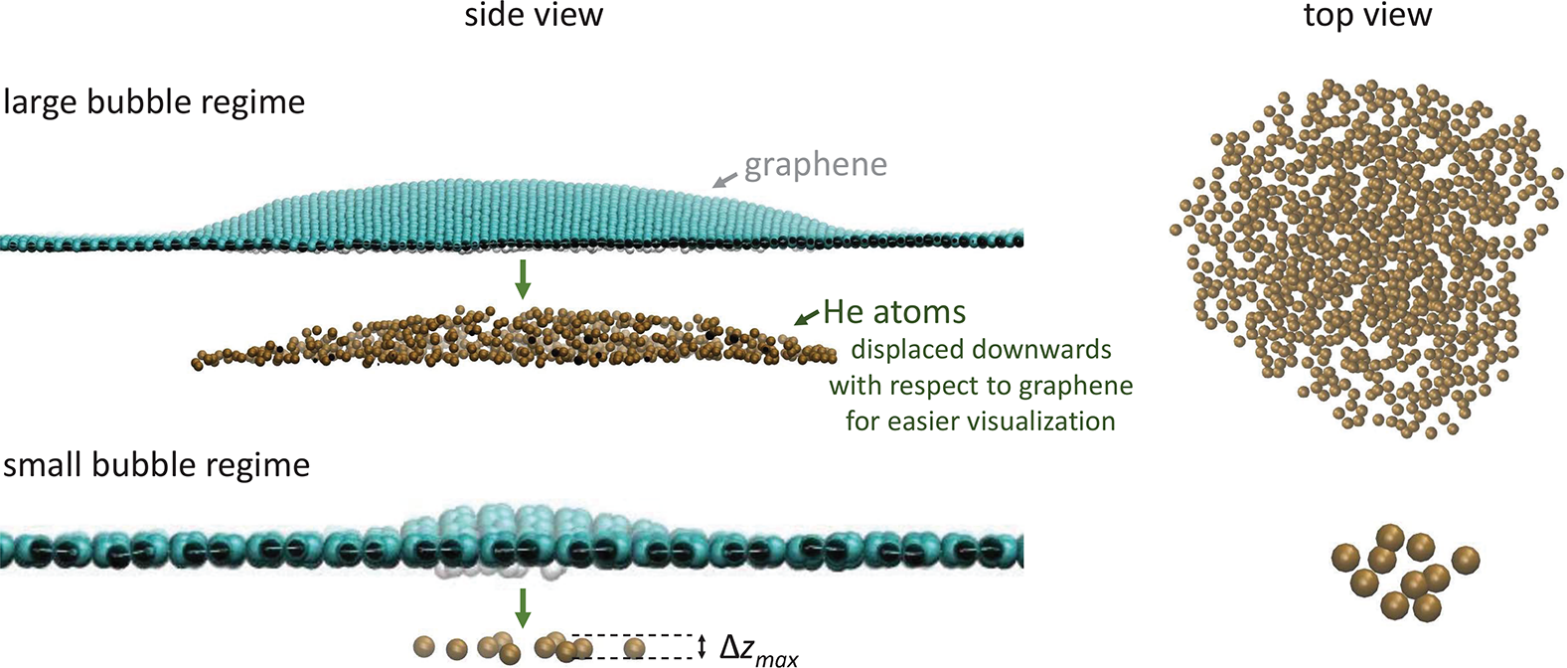
Top and side view of examples of large and small He bubbles, simulated using MD. The He atoms are shown displaced downward, away from the graphene layer, for easier visualization. In the small-bubble regime, the He atoms are distributed in a monolayer-like configuration (i.e., without being on top of each other) but still with a significant out-of-plane motion amplitude (Δ*z*_max_).

This behavior is well reproduced by our MD simulations of bubbles with a varying number of trapped atoms (from 800, with *R* of a few nm, down to a few atoms, with *R* below 1 nm—Figure 2). In particular, for the smallest bubbles with a small number of trapped atoms (of the order of 10), the monolayer-like configuration is clearly observed in our MD simulations (Figure 3), while for the larger bubbles, the trapped atoms are distributed over multiple layers of gas atoms (Figure 3). The significant spread in aspect ratio for a given radius (experimental data points in Figure 2) is likely due to a varying strength of the adhesion between graphene and the Pt surface (γ_GS_) over the sample surface. Such nonhomogeneity can result from the varying (relative) orientation of the graphene and Pt lattices (the graphene layers grown on Pt are polycrystalline—Figure S8) as well as possible local variations in graphene–Pt adhesion due to the subtle graphene disorder observed in the STM and Raman data, discussed above. Although this possible effect of subtle disorder on the graphene–substrate adhesion may also play a role in the stability of the bubbles, it does not appear to be a dominant effect, since our MD simulations reproduce well the stability for Pt and instability for Cu without taking into account this disorder.

## Dependence on Trapped Element

Let us first consider the *R* ≫ 1 nm regime, where the universal scaling given by eq 1(5) applies, and thereby extract γ (given in Table 1) for each gas (He, Ne, and Ar). Although the values of  for *R* ≫ 1 nm for He, Ne, and Ar are different, the spread over the various bubbles (reflected in a large standard deviation) blurs out these differences. Nevertheless, the data strongly suggest that this quantity does depend on the trapped element. Such a scenario can be understood as due to a variation in γ, that is, higher for Ne (γ ≈ 0.13 eV/Å^2^) and for He (∼0.08 eV/Å^2^) than for Ar (∼0.02 eV/Å^2^). Taking γ_GS_ = 0.25 eV/Å^2^ for graphene on Pt^23^ implies that γ_Sb_ + γ_Gb_ is of the order of γ_GS_ for Ar (giving γ = 0.02 eV/Å^2^) but significantly smaller for He and Ne. In other words, in the bubble configuration, the interaction (of vdW nature) of the gas atoms with the Pt surface or with the graphene layer appears to be more repulsive for Ne and He compared to Ar.

**Figure 4:**
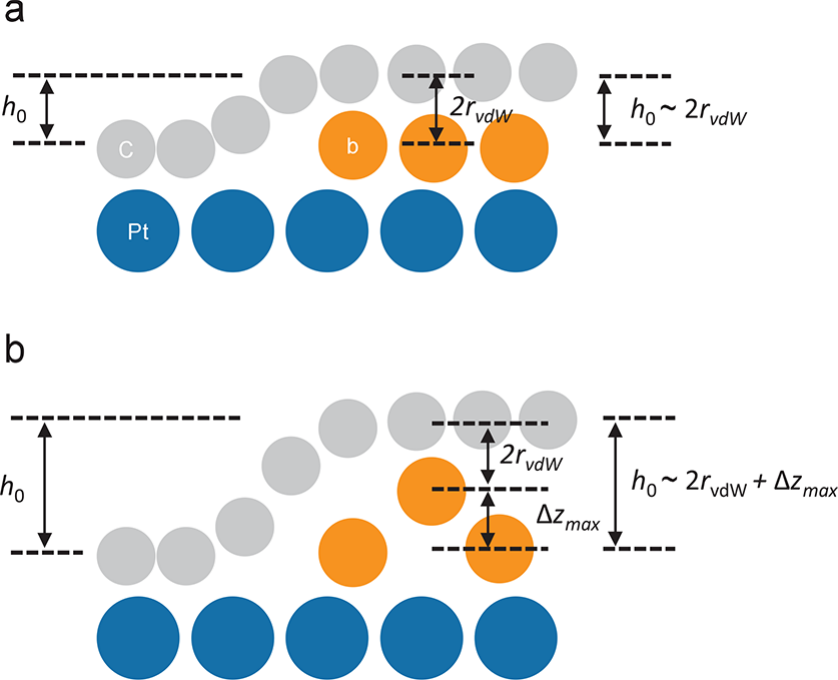
Schematics illustrating the relation between the bubble height in the small-bubble limit (*h*_0_) measured with STM, the vdW diameter of the trapped atoms (2*r*_vdW_), and the maximum out-of-plane motion amplitude (Δ*z*_max_): (a) When Δ*z*_max_ ≈ 0, *h*_0_ ≈ 2*r*_vdW_ and (b) when Δ*z*_max_ > 0, *h*_0_ ≈ 2*r*_vdW_ + Δ*z*_max_.

A similar trend is observed in the low-*R* regime, where the Ne bubbles clearly reach higher *h*_max_ values than for He and Ar bubbles (Figure 2) and *h*_max_^0^ is also larger (although with a significant spread over various bubbles) for Ne than for He and Ar (Table 1). This is particularly noteworthy, as it does not follow the same trend as the vdW diameter (2*r*_vdW_), which increases from He, to Ne, to Ar (Table 1). Since, to a first approximation, one would expect the height of a bubble filled with a monolayer of noble gas atoms to scale with the vdW diameter of those atoms, other factors must also be playing a role, namely differences in atomic compressibility β and in out-of-plane motion of the noble gas elements. The effect of atomic compressibility is particularly obvious considering that while *h*_max_^0^ is approximately equal to 2*r*_vdW_ for He (2.9 and 2.86 Å, respectively) and only slightly higher for Ne (3.5 and 3.16 Å), it is significantly smaller for Ar (3.1 and 3.88 Å). This is indeed consistent with the fact that Ar is the most compressible of the three elements, followed by He and Ne (Table 1, from ref (22)). In addition to the compressibility, the differences in magnitude of the out-of-plane motion of the gas atoms are likely to also play a role, in particular, since as mentioned above for Ne, *h*_max_^0^ is even larger than 2*r*_vdW_ (3.5 and 3.16 Å, respectively). This is indeed consistent with our MD simulations. The average *z*-motion amplitude (averaged over time and over the trapped atoms) obtained from the MD simulations (Figure 3), for the smallest bubbles (⟨Δ*z*⟩ in Table 1) is indeed significantly larger for Ne than for He and Ar. This out-of-plane motion forces *h*_max_^0^ to be larger than the (compressed) vdW diameter (Figure 4b), that is, larger than that associated with a rigid atomic monolayer (Figure 4a), by an amount Δ*z*_max_ that depends on γ_Sb_ and γ_Gb_. In other words, the weaker the binding of the trapped atoms to the graphene layer and to the Pt surface, the more the gas atoms are allowed to move out-of-plane, and therefore, the more the sub-nm bubbles deviate from a static monolayer of (compressed) noble gas atoms.

## Extreme Strain and Pressure

**Figure 5:**
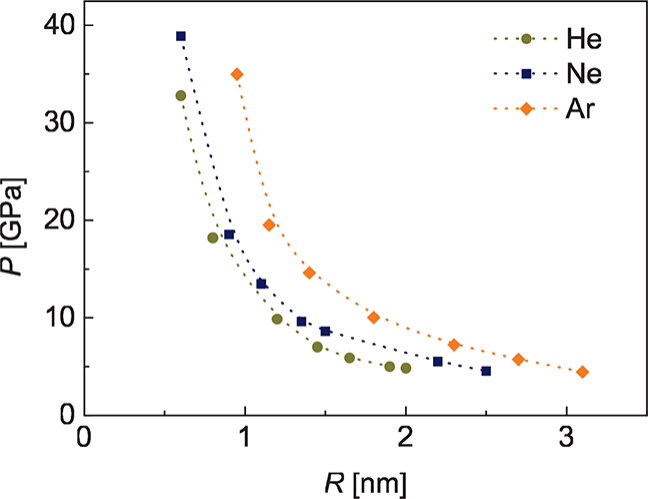
Pressure estimated from the MD simulations for He, Ne, and Ar bubbles in graphene/Pt(111), as a function of bubble radius. The lines are guides to the eye.

The breakdown of the universal scaling, leading to extreme aspect ratios, is likely to be associated with other unusual physical properties in these subnanometer bubbles. Although studying such properties in detail is beyond the scope of this Letter, it is worthwhile discussing strain and pressure as examples. The tensile strain induced on graphene by the underlying trapped atoms can be estimated from our STM measurements as follows. From the STM topography of a bubble, one can determine the surface area of the graphene layer that wraps the three-dimensional bubble (*A*_surface_) as well as the (projected) area of the base of the bubble (*A*_projected_). *A*_surface_ is the area of the strained graphene region, whereas *A*_projected_ would be the area of that region if the bubble would not exist. The tensile strain can then be estimated as . An accurate estimate requires smooth, low-noise, atomic-resolution STM micrographs of single bubbles. From selected high-quality micrographs of two He bubbles with *R* ≈ 1 nm, we obtain ϵ_A_ values of the order of 10%. More details are provided as Supporting Information. Regarding pressure, according to the general understanding of surface-induced pressure in solids, it scales with the ratio of surface area to the volume of the solid phase.^24^ For the bubbles under consideration here, as *R* decreases and the atoms inside the bubbles become more monolayer-like, the surface-to-volume ratio (∼Δ*z*^–1^) increases dramatically, since Δ*z* → 0. One can therefore expect the pressure to also increase dramatically in the limit of small *R*. Our MD calculations show exactly that (Figure 5), that is, a diverging behavior with decreasing *R*, reaching remarkably high values of up to ∼30 GPa. These values were obtained using the stress-tensor-based method,^25^ as recently applied to nanobubbles in graphene,^6^ with pressure being given by
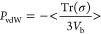
2where *P*_vdW_ is the vdW pressure, Tr(σ) is the trace of the virial stress tensor, and *V*_b_ is the volume available to the gas atoms. We note that this method, based on the virial stress tensor, is more general and more appropriate in the present case compared to other methods based on membrane theory and plate theory. The latter methods are based on elasticity theory, which is valid in the large-bubble limit, but tends to overestimate the pressure for small bubbles.^6^ At such high pressures, at room temperature, these noble gases are expected to be in a solid phase or near their melting transition, which is around 10 GPa for He,^26^ 5 GPa for Ne,^27^ and 1.5 GPa for Ar.^28^ Considering the pressures estimated here (Figure 5), one would then expect Ne and Ar to be in a solid-like phase, while He, with the highest melting transition (10 GPa), is expected to behave more liquid-like (possibly near a solid-like phase for *R* < 1 nm). Our MD simulations are indeed consistent with this expectation (cf. the videos provided as Supporting Information), showing rather stable ordered atomic arrangements for Ne (Videos V1 and V2) and Ar (V3 and V4) and more disordered and dynamic arrangements for He (V5 and V6).

## Stability on Pt versus Instability on Cu

As mentioned above, unlike for Pt, bubbles are not observed on Cu flat terraces. It appears that only the atoms that are trapped in defects (e.g., dips and terrace edges, as shown in Figure S1) are immobilized as intercalated species. The remainder is likely to escape via graphene defects (e.g., holes). This bubble instability for graphene on Cu is confirmed in our MD simulations (cf. the videos provided as Supporting Information): If a bubble configuration (similar to those in Pt—videos V7 and V8) is given as the initial state, the time evolution shows graphene peeling off the Cu surface, resulting in the dispersion of the trapped gas atoms (V9 and V10). This instability can be easily understood as due to the much weaker adhesion of graphene to Cu (γ_GS_ = 0.045 eV/Å^2^^29^) compared to Pt (0.251 eV/Å^2^^23^), that is, the Cu–graphene binding is too weak to sustain the high pressures associated with the bubbles. In order to assess if the gas–metal adhesion (γ_Sb_) also plays a role in this stability difference, we used DFT to calculate the adsorption energy and the adsorption distance of isolated He, Ne, and Ar atoms on Pt(111) and Cu(111) surfaces (Table S4). Although the adsorption energies are indeed larger for Pt than for Cu when comparing the gas elements one by one, it still does not explain the observed difference in stability. For example, the adsorption energies of Ar on Cu (for which bubbles are not stable) are larger than those of He on Pt (for which stable bubbles are observed). We therefore conclude that bubbles are not stable on flat Cu terraces due to the much weaker adhesion of graphene to Cu as compared to Pt.

To conclude, using ULE implantation of noble gas ions (He, Ne, and Ar), we produced nanobubbles on graphene with varying radius, from few nm down to subnanometer scales. These nanobubbles are stable for graphene on Pt but not for graphene on Cu. While the bubble aspect ratio behaves differently for the different elements, the universal scaling behavior (that was previously established for larger bubbles) breaks down in all three cases, for a bubble radius around 1 nm, as the bubble height approaches a minimum corresponding to about an atomic monolayer. We interpret the observed dependencies on the substrate and trapped element in terms of the adhesion energies between the three constituents: graphene, the substrate, and the trapped noble gas element. Moreover, these nanobubbles are found to induce high levels of strain (of the order of 10%) on the overlaying graphene. In addition to providing insight on the spatial distribution of the trapped atoms and its relation to the bubble morphology and stability, molecular dynamics calculations also allowed us to estimate the vdW pressure inside the bubbles, exceeding 30 GPa for the smallest bubbles. These remarkably high strains and pressures illustrate the unique characteristics of this subnanometer bubble regime (achievable using ultralow energy ion implantation) compared to the previously studied (larger) nanobubbles. These unique properties offer new opportunities, for example, to study physical states of matter and chemical reactions under high (vdW) pressure or electronic phenomena associated with strain-induced pseudomagnetic fields in graphene. Since the behavior reported here is largely determined by the adsorption energies between the three constituents (2D material, substrate, and trapped substance), one can expect similar behavior for other 2D materials (e.g., transition metal dichalcogenides such as MoS_2_), which expands even further the range of possible applications. In particular, since the bubble formation is based on ion implantation, our approach is compatible with virtually any implanted element, 2D material, and substrate.

## Supporting Information Available

Ne atoms in small bubble on Pt (bottom view) (MP4)
Ne atoms in large bubble on Pt (bottom view) (MP4)
Ar atoms in small bubble on Pt (bottom view) (MP4)
Ar atoms in large bubble on Pt (bottom view) (MP4)
He atoms in small bubble on Pt (bottom view) (MP4)
Small He bubble on Pt (MP4)
He atoms in large bubble on Pt (bottom view) (MP4)
Large He bubble on Pt (MP4)
Small He bubble on Cu (MP4)
Large He bubble on Cu (MP4)
(1) Methods: details on the growth of graphene on Cu and Pt (by chemical vapor deposition), the ultralow energy ion implantation, the experimental characterization (scanning tunneling microscopy, Raman spectroscopy, low-energy electron diffraction), and computational methods (molecular dynamics simulations and density functional theory calculations); (2) additional results from calculations and from experimental characterization, with accompanying references.^30−63^ (PDF)
