# Supplementary material for: Breakdown of Universal Scaling for Nanometer-Sized Bubbles in Graphene
Source: Nano Lett. 2021 Sep 14;21(19):8103–10. doi: 10.1021/acs.nanolett.1c02470 (PMC9286314; doi:10.1021/acs.nanolett.1c02470)
Supplement: Supplementary file 11 — nl1c02470_si_011.pdf [file nl1c02470_si_011.pdf]

Breakdown of Universal Scaling for Nanometer-Sized Bubbles in Graphene

Renan Villarreal,[*,†,††] Pin-Cheng Lin,[†,††] Fahim Faraji,[‡,¶] Nasim Hassani,[§] Harsh Bana,[†] Zviadi Zarkua,[†] Maya N. Nair,[‖] Hung-Chieh Tsai,[⊥,#] Manuel Auge,[@] Felix Junge,[@] Hans C. Hofsaess,[@] Stefan De Gendt,[⊥,#] Steven De Feyter,[△] Steven Brems,[⊥] E. Harriet Åhlgren,[∇] Erik C. Neyts,[‡] Lucian Covaci,[¶] François M. Peeters,[¶] Mehdi Neek-Amal,[*,§] and Lino M. C. Pereira[*,†]

†*Quantum Solid State Physics, KU Leuven, 3001 Leuven, Belgium*

‡*Research group PLASMANT, Department of Chemistry, Universiteit Antwerpen (UIA), 2610 Antwerpen, Belgium*

¶*Departement Natuurkunde, Universiteit Antwerpen (UIA), 2610 Antwerpen, Belgium*

§*Department of Physics, Shahid Rajaee Teacher Training University, Lavizan, Tehran, Iran*

‖*CUNY Advanced Science Research Center, 85 St. Nicholas Terrace, New York, New York 10031, USA*

⊥*imec vzw (Interuniversitair Micro-Electronica Centrum), 3001 Leuven, Belgium*

#*Department of Chemistry, Division of Molecular Design and Synthesis, KU Leuven, 3001 Leuven, Belgium*

@*II.Institute of Physics, University of Göttingen, 37077 Göttingen, Germany*

△*Department of Chemistry, Division of Molecular Imaging and Photonics, KU Leuven, 3001 Leuven, Belgium*

∇*Faculty of Physics, University of Vienna, 1090 Vienna, Austria*

††*R.V. and P.-C.L. contributed equally to this work*

E-mail: renan.villarreal@kuleuven.be; mehdi.neekamal@gmail.com; lino.pereira@kuleuven.be

Methods

CVD growth of graphene

Our samples consist of epitaxial graphene grown by chemical vapor deposition (CVD) on epitaxial Pt(111) and Cu(111) thin films grown on sapphire(0001) substrates.[16,17] 2 inch polished Czochralski grown sapphire wafers cut along c-plane with <0.1° miscut are used to prepare Al_2O_3 template wafers. The as-received wafers are first cleaned in a 3:1 volume ratio of the acid mixture of H_2O_4:H_3PO_4 at 180 °C for 20 min, followed by an ultra pure water rinse for 3 min. Immediately after the cleaning process Pt and Cu films are grown. For Pt, a 500 nm thick film is evaporated on the sapphire wafer by electron-beam deposition (Pfeiffer PLS 580) while keeping the temperature of the wafer at approximately 550 °C on a heated chuck. The base pressure is 4×10^{-7} mbar. The deposition rate is controlled at 1 Å s^{-1} by a quartz crystal rate monitor. For Cu films, the cleaned sapphire wafers are placed in a Nimbus 310 sputtering setup with a base pressure of 4×10^{-6} mbar. Substrates are placed using a 200 mm Si pocket wafer and sputtering is performed at room temperature for 173 s (21 passes under target) at 6×10^{-3} mbar Ar pressure. The Cu deposition rate is calibrated and corresponds to a rate of about 29 Å s^{-1}. The applied power was 3000 W and the throw distance is approximately 50 mm. The growth of CVD graphene on the Pt films is performed in a cold wall CVD reactor (Aixtron Black Magic 6 inch) at a pressure of 750 mbar, a temperature of 1100 °C, and with a gas mixture of 9 sccm CH_4 and 800 sccm H_2. Before and after the 40 min growth period, the reactor is ramping up and cooling down in a H_2 environment. The graphene growth process on the Cu films has been optimized taking into account oriented monolayer growth and low Raman D peak. The growth conditions are 5000 sccm Ar: 125 sccm H_2: 0.3 sccm CH_4 at a pressure of 750 mbar close to the melting point of Cu. The growth time is 30 min, while ramp-up and down is performed in a mixture of Ar and H_2.

Ultra-low energy ion implantation

Ultra-low energy (ULE) ion implantation was performed by electrostatic deceleration of the ion beam ($^{4}He^{+}$, $^{22}Ne^{+}$, $^{40}Ar^{+}$) from 30 keV to 25 eV, with perpendicular incidence with respect to the sample surface, to a fluence of 1×10^{15} ions per cm^2 (fluence rate of the order of 2×10^{12} cm$^{-2} \cdot$ s^{-1}) measured by integration of the electric current on sample during implantation.[18–20]

Scanning tunneling microscopy

The STM micrographs reported (and used for the analysis) here were obtained in UHV (base pressure $\sim 10^{-10}$ mbar) at room temperatures using an Omicron LS SPM and an Unisoku USM1000. We used cut Pt-Ir tips. All topographies were acquired in constant $I_{tun.}$.

Raman spectroscopy

Raman spectra were measured using a confocal Raman microscope (Monovista CRS+, S&I GmbH) equipped with a 532 nm Nd:YAG laser. The laser was directed onto the sample surface through an objective (OLYMPUS, BX43 50×, numerical aperture 0.75), with the maximum laser power remaining below 1 mW in order to avoid laser-induced modification. All the measurements were obtained in ambient conditions, at room temperature.

Low-energy electron diffraction

The as-grown graphene/Pt(111) was characterized by a rear view AEP 8011 (VG Scientific) LEED setup. LEED images were recorded using the screen voltage and an electron beam spot of 1 kV and 1 mm, respectively. No thermal cleaning treatment was performed before the measurements.

Molecular dynamics: ULE ion implantation

The simulated system consists of a 4.0×3.5 nm relaxed freestanding graphene sheet. Periodic boundary conditions are applied in x and y directions with Berendsen temperature control[50] at a few atomic rows at the edges to mimic energy dissipation into an infinite system. The C-C interactions are modeled with a bond order potential by Brenner *et al.*[51] smoothly joined to the universal repulsive potential[52] at small atomic separations. The He/Ne/Ar-C interactions are modeled by the universal repulsive potential.[52] The projectile is set at a distance of 6 Å above the surface and given the respective energy (10, 15, 20, 25, 30, 40 eV) along the normal of the surface. A total of 300 impacts was calculated for each atom and energy. The impact points are generated randomly within an irreducible area of the lattice (one twelfth of a hexagon). The simulation time is set for 10 000 fs providing enough time for the structure to find a local energy minimum after the impact. The time step is scaled according to the fastest moving particle in the system.

Molecular dynamics: Bubbles

We simulated the nanobubbles using molecular dynamics (MD). The simulation system comprised a graphene sheet spanned over Pt(111) and Cu(111) substrates, between which a varying number (from 6 to 800 atoms) of noble gas atoms (He, Ne and Ar) were inserted. The graphene sheet dimensions were 7.5 × 7.5 nm^2 and at the beginning, the middle region of the graphene was manually lifted to form an artificial bubble so that the trapped atoms could be accommodated. The substrate and the graphene sheet were modeled using the embedded atom method (EAM)[30] and adaptive intermolecular reactive bond order (AIREBO) potential[31] while the trapped materials, as well as the interactions among the substrate, graphene sheet and trapped materials, were modeled using the Lennard-Jones (LJ) 12-6 potential. The LJ parameters were set according to ref. 32 for the trapped gases, ref. 33 for C and ref. 34 for Pt and Cu, and the cross parameters were calculated using Kong's formula.[35] The van der Waals interactions modeled by LJ-potentials were cut off at 10 Å distances.

The energy of the system initially minimized using conjugate-gradient algorithm, followed by gradually heating the system from frozen to room temperature using Nose-Hoover thermostat.[61,62] Next, the NVT simulation at room temperature was continued until the system exhibited steady state after which the quantities of interest were calculated. The MD simulations were carried out using the Lammps package[36] with the Velocity-Verlet discretization[63] of the Newtonian equation of motion and 1 fs time-step. The average out-of-plane motion amplitudes ($\langle \Delta z \rangle$) for the smallest bubbles was obtained by time-averaging among several MD configurations in equilibrium.

The pressure inside the bubbles was calculated using the stress formulation.[25]

Density functional theory calculations

The adsorption of the noble gases on Pt(111) and Cu(111) surfaces was assessed using density functional theory calculations. The DMol3 module in the Materials Studio software was used to perform all calculations.[37] The Perdew-Burke-Ernzerhof (PBE) functional[38] with the generalized gradient approximation (GGA) was adopted in conjugation with the double numerical plus polarization (DNP) basis set for the calculations. The Tkatchenko-Scheffler van der Waals method with dispersion-corrected density functional theory (DFT-TS)[39] was performed to precisely characterize the adsorption and penetration behavior of gases. The global orbital cutoff and the self-consistent field (SCF) convergence threshold were set to be 0.6 nm and 10^{-6} a.u., respectively. To avoid the interaction of two adjacent layers, a vacuum thickness of 1.5 nm was inserted in the z direction. The optimized crystal lattices (a, b) of all the studied graphene monolayers was set to 1.727 nm.

Additional results from the calculations and from experimental characterization

Absence of bubbles for graphene on Cu(111)

As shown in Fig. S1, contrary to graphene on Pt(111), graphene on Cu(111) (implanted with the noble gases) did not exhibit bubbles on the flat terrace surfaces. Only some bubbles/wrinkles were sporadically observed on some terrace edges and surface defects. These rare bubbles/wrinkles are associated with intercalated noble gas atoms which diffuse (as intercalated atoms) and become trapped in topographic defects. Since these bubbles/wrinkles are rarely observed, we conclude that this trapping is less efficient than the loss of gas atoms via graphene defects (e.g. holes), that is, that the majority of the implanted atoms are not trapped in such defects, but instead escape through graphene defects.

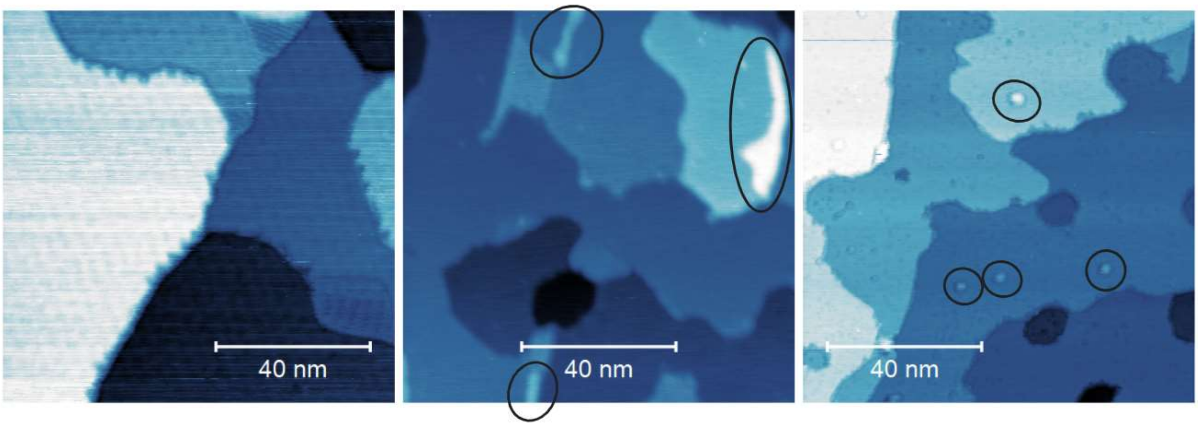

Figure S1: STM topographies of (left) pristine graphene on Cu(111) and helium implanted graphene on Cu(111) exhibiting: (middle) bubble/wrinkles in terrace edges and (right) bubbles trapped in dip-like defects on the Cu surface. These bubbles/wrinkles are indicated by black ellipses.

Transmission and vacancy formation

Figure S2 summarizes the results from the MD simulations that we performed in order to select the implantation energy. Transmission probability is defined as the number of events in

which the impinging atom is found to go through the graphene layer divided by the number of simulated events. The vacancy formation probability is defined as the number of events that produce a stable vacancy divided by the number of simulated events. According to our simulations (Fig. S2), an implantation energy of 25 eV provides significant transmission (of the order of 50%) while avoiding vacancy formation. We note however that these simulations are used merely as a guideline. The effective intercalation efficiency (fraction of implanted atoms that are stopped between the graphene layer and substrate) depends not only on the transmission but also on eventual backscattering from the substrate (which is not taken into account in our simulations). Moreover, the defect creation dynamics is likely to be more complex due to the presence of the Pt/Cu substrate, as can be inferred from the fact that our Raman measurements show the onset of disorder even for an implantation energy of 25 eV.

Pristine graphene on Pt(111)

The surfaces of pristine and He-implanted graphene on Pt(111) are compared in Fig. S3. The bubbles produced by intercalation of noble gases after implantation (right panel) can be easily identified in comparison to non-implanted (pristine) graphene that exhibits a flat surface with a negligible amount of nanometer sized protrusions (*e.g.,* contaminants, bubbles, etc.). The comparison of the pristine surface with the as-implanted surface at the atomic scale (in the insets) reveals that the mostly homogeneous moiré superstructure is mildly affected by disorder introduced by the implantation process.

Determination of the structural properties of the bubbles

The structural properties of the bubbles are extracted experimentally from STM topographies by the height-based masking process provided by the open source software Gwyddion which allows to select all bubbles from a single terrace area of the surface. The area is leveled by mean plane subtraction, rows are properly aligned and the minimum is set to zero.

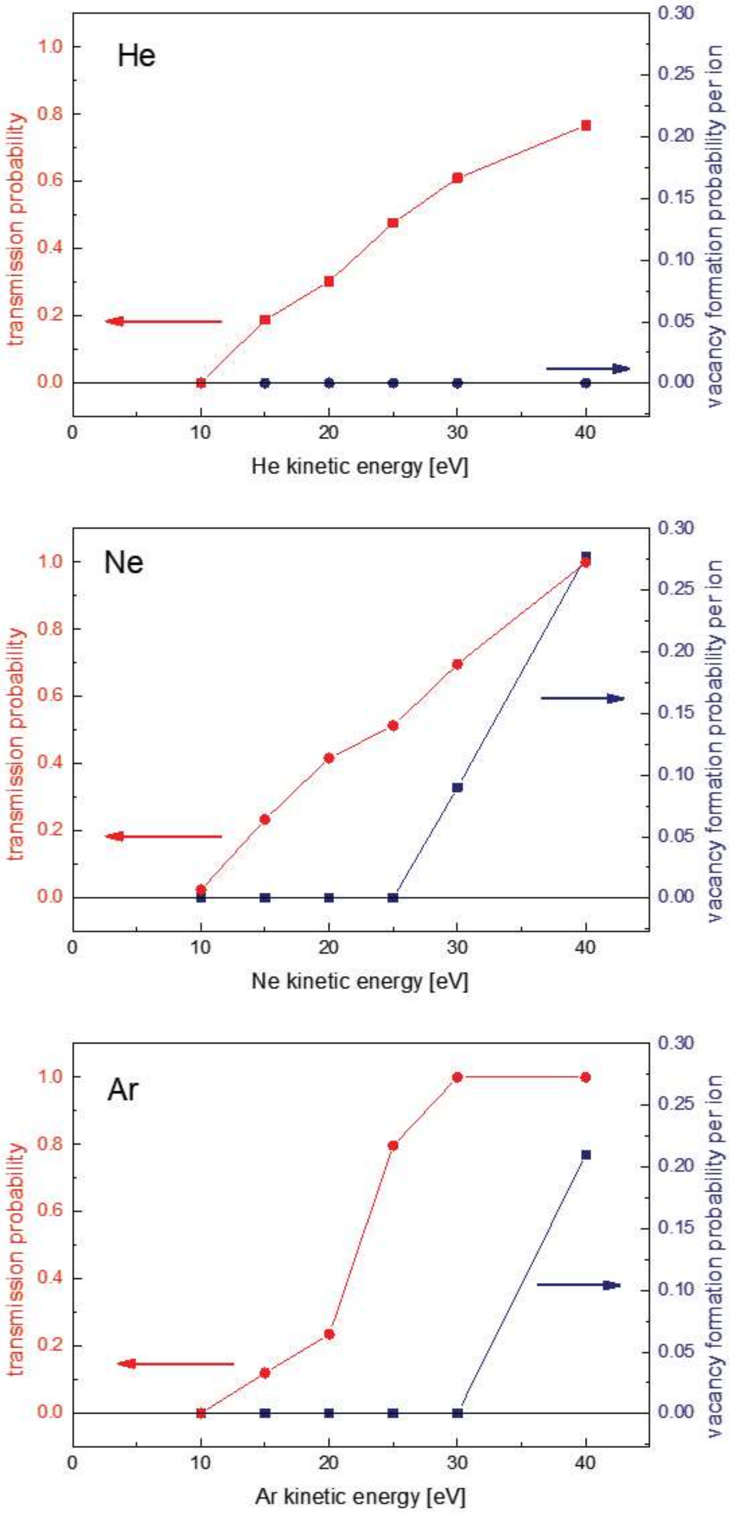

Figure S2: Transmission probability and C-vacancy formation probability, simulated using MD, as a function of kinetic energy, for the three gas elements (He, Ne, Ar), for suspended graphene.

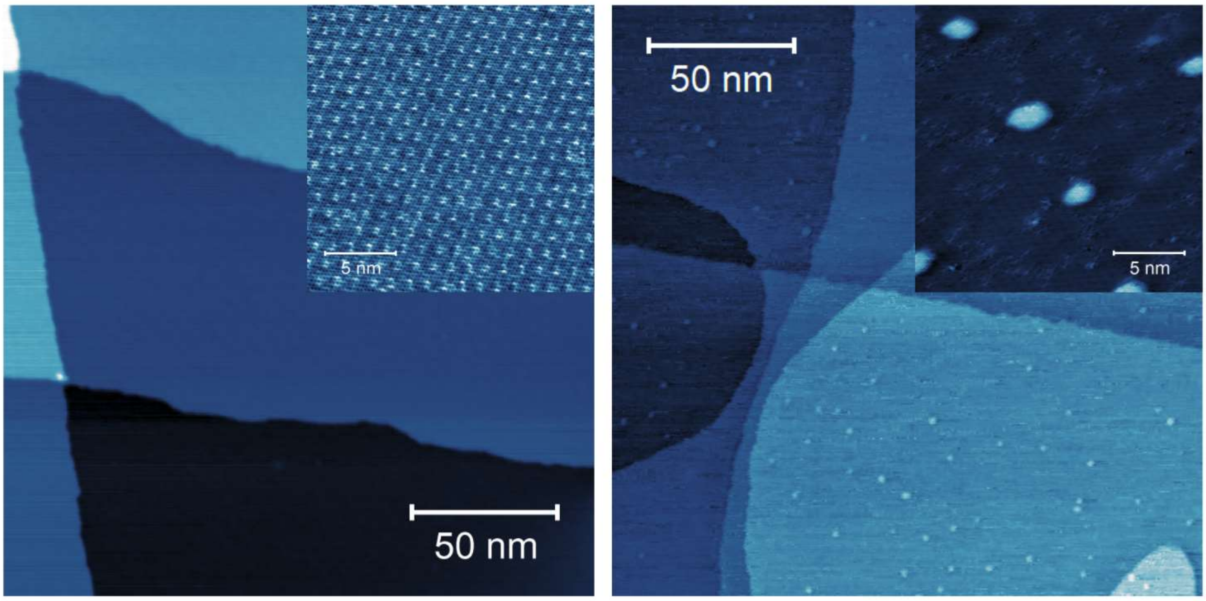

Figure S3: STM topographies of (left) pristine graphene on Pt(111) and (right) helium implanted graphene on Pt(111) with insets showing the moiré superstructure and graphene lattice for both cases.

Then, the mask is applied to the area by using the height distribution as a reference. In figure S4(a,b), we show an example of a topography with its corresponding height distribution. As shown in figure S4(c), the mask can be applied to select only (mostly) objects on the surface that correspond to bubbles. We cannot discard that some of the objects selected may be something different from a bubble but we expect this to be a small percentage. The projected base radii from all of the bubbles selected are extracted assuming an equivalent bubble disc shape (this is an approximation as not all bubbles are perfectly circular). The maximum heights are also extracted based on the bubble selection and (when necessary) corrected for the height offset introduced by surface defects exhibiting lower height than the terrace (e.g., those highlighted in figure S4(d)). This offset is determined based on the maximum (counts) of the height distribution which corresponds to the terrace height. After the latter, we obtain a list of radii and heights per topography. We filter the data set by their radii in two steps to discard outliers. First, we set a radius lower limit as 5 Å and ignore anything below that threshold assuming that they mostly correspond to defects, con-

taminants or lattice distortions resulting from the implantation process. Second, we reset the radius lower limit per list (topography) to minus one standard deviation of the average radius value (assuming a Gaussian distribution), therefore removing those bubbles that are farther than one standard deviation below the average radius.

Additional data on larger Ar bubbles

Given the small number of bubbles in the large-radius regime (around 3 nm and larger) identified in the samples described in the main text, in this section we provide additional data that further supports the notion that the aspect ratio converges to a constant value with increasing radius, i.e. entering the universal scaling regime. Figure S5 shows, in addition to the data on Ar bubbles presented in the main text, additional data points corresponding to Ar bubbles identified in a sample prepared with a slightly different procedure, namely with a different implantation energy (15 eV). Given the low amount of data, no significant conclusions can be drawn at the moment regarding the effect of changing these preparation parameters; further studies are ongoing. Nevertheless, the data does confirm that the aspect ratio converges to a constant value with increasing radius, i.e. entering the universal scaling regime, as discussed in the main text.

Effect of bending rigidity

In ref. 5, the universal scaling (equation 1 in our main text) is derived under the assumption that the elastic energy associated with the out-of-plane bending rigidity is much smaller than that associated with the in-plane stiffness, and can therefore be neglected. This is a valid assumption for bubbles with dimensions significantly larger than $\sqrt{Y/\kappa} \sim 0.4$ nm, where κ is the bending rigidity.[5] However, in the present case, since the bubbles are small enough to possibly enter this new regime, it is important to evaluate whether the bending rigidity starts playing a significant role. In order to do that, we reintroduce the bending energy term:

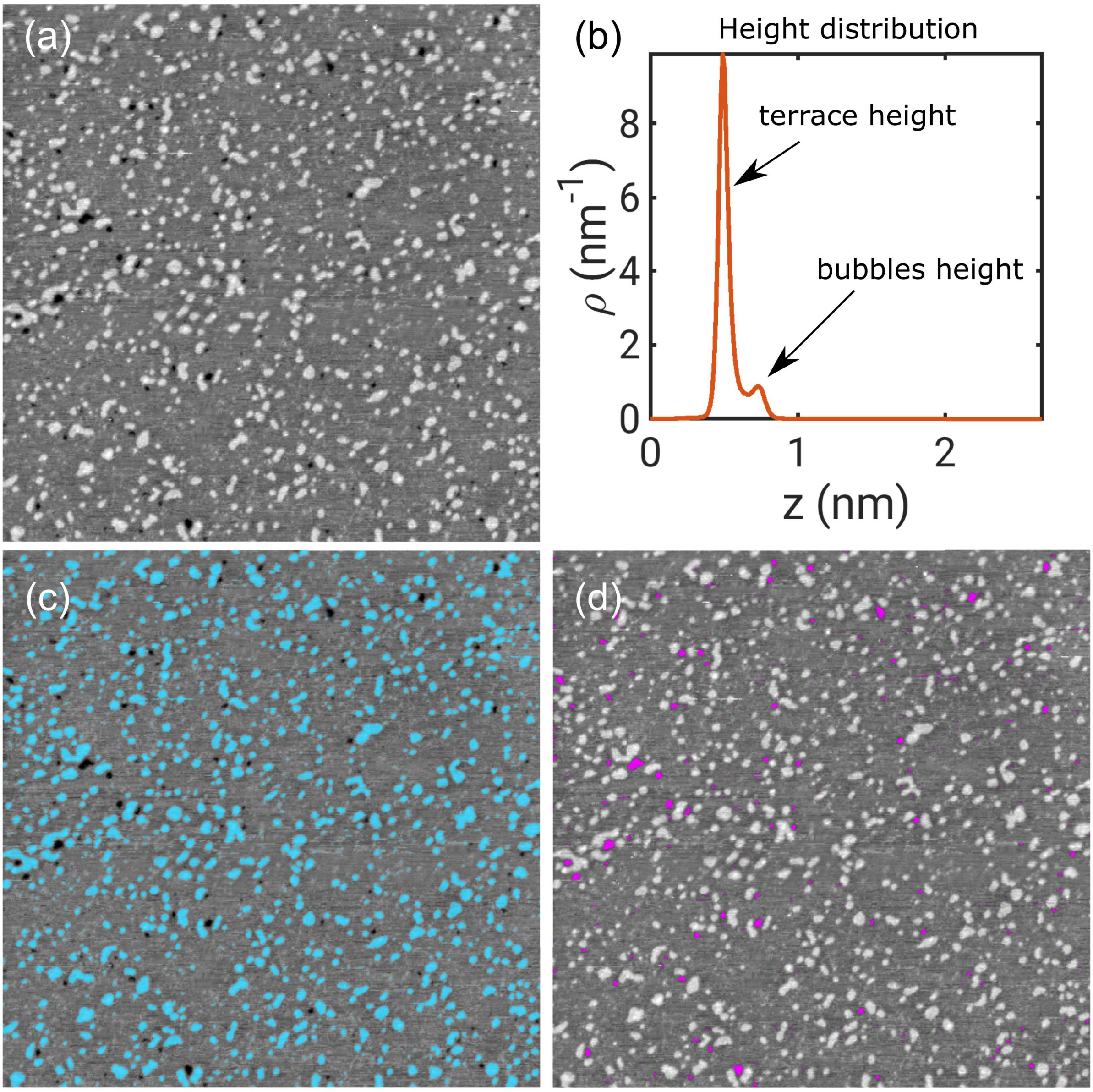

Figure S4: (a) STM topography of argon bubbles of graphene on Pt(111). (b) Height distribution plot of the topography showing a peak corresponding to the terrace height which is offset from zero due to surface defects exhibiting lower heights and another peak corresponding to the bubbles height. (c) STM topography from (a) with a mask applied on the bubbles. (d) STM topography from (a) with a mask applied on the surface defects exhibiting lower height than the terrace.

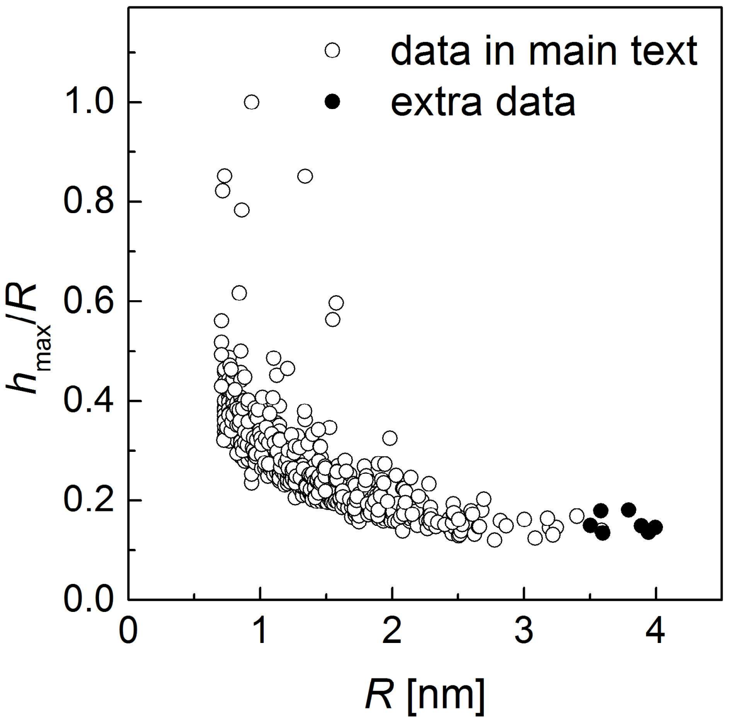

Figure S5: h_{max}/R as a function of R obtained from STM micrographs for Ar bubbles. The open circles correspond to the data presented in the main text (Fig. 2 (c)). The filled circles correspond to additional data, i.e. larger bubbles identified in samples with slightly different preparation parameters. Each data point corresponds to one bubble.

$$E_{\rm bend} = c_3 \kappa \frac{h_{\rm max}^2}{R^2} \tag{3}$$

in the expression for the total energy in ref. 5, where c_3 is a constant of the order of unity. Doing so, changes equations 6 in ref. 5 as follows:

$$\begin{aligned} c_1 Y \frac{4h_{\rm max}^3}{R^2} - c_V R^2 P + 2c_3\kappa \frac{h_{\rm max}}{R^2} = 0 \\ -c_1 Y \frac{2h_{\rm max}^4}{R^3} + 2\pi\gamma R - 2c_V h_{\rm max} RP - 2c_3\kappa \frac{h_{\rm max}^2}{R^3} = 0, \end{aligned} \tag{4}$$

where we also neglected the effect of residual strain induced by the substrate, as in ref. 5. By eliminating P, we obtain:

$$5c_1 Y h_{\rm max}^4 + 3c_3\kappa h_{\rm max}^2 - \pi\gamma R^4. \tag{5}$$

Solving for $h_{\rm max}^2$ gives:

$$h_{\rm max}^2 = \frac{\sqrt{9c_3^2\kappa^2 + 20\pi c_1 Y \gamma R^4} - 3c_3\kappa}{10c_1 Y}. \tag{6}$$

The aspect ratio $h_{\rm max}/R$ is no longer independent of R: it decreases with decreasing R, as one would expect, since the bending rigidity acts to make graphene flatter, i.e. decreasing the bubble height. The effect is negligible for large R, consistent with neglecting the effect of bending rigidity for large bubbles, and increases with decreasing R and decreasing γ. Taking the parameters describing the present bubbles, and in particular taking $\kappa = 1.46$ eV,[57] and the values of R and γ that would maximize the effect of bending rigidity, i.e. smallest R (~ 0.5 nm) and smallest γ (~ 0.02 eV Å^{-2}), decreases the aspect ratio $h_{\rm max}/R$ by only a few percent. We can then conclude that the effect of bending rigidity on the aspect ratio of our bubbles is negligible, and in fact tends to decrease the aspect ratio when compared to the universal scaling expression, not increase it as observed by us here. Therefore, a different

mechanism must be responsible for the observed increase, as described in the main text.

Quantitative analysis of Raman spectroscopy

Raman spectroscopy is commonly used to characterize the disorder in graphene.[40–43] Although the selected implantation energy is below the threshold for vacancy formation (Fig. S2), Raman characterization shows some degree of disorder for Ne and even more for Ar (Fig. S6). This disorder is likely associated with the breaking of C-C bonds without the production of C vacancies. Such damage has been reported for irradiation with laser light and low-energy electrons,[53–56] which also does not produce vacancies but does lead to the appearance of a D band and the modification of the G and 2D bands in the Raman data. Such effects are also consistent with the perturbed periodicity of the atomic lattice observed in the STM data (Figs. 1d,e). In figure S6, the presence of D band (originating from the disorder scattering in sp^2 carbon, the so-called breathing mode of the graphene lattice, which is an intervalley scattering process) was observed for Ne and Ar but not for He. Moreover, except for He implanted graphene, the D' band (intravalley scattering process induced by disorder, located at slightly higher Raman shift than the G band) appears in the samples implanted with Ne and Ar, in both substrates. In the case of Gr/Pt(111), the D' band cannot be distinguished from the G band because of two reasons: (1) peak broadening induced by disorder; (2) suppression of the band intensity caused by the Pt substrate. In contrast, for Gr/Cu(111), although the disorder induced peak broadening is also expected, the substrate induced suppression is less prominent, and the D' band is therefore clearly distinguishable from the G band. It is known that the intensity of the graphene signature bands, G and 2D (also referred to as G' in literature) bands, are suppressed by the metallic substrate. The strength of the suppression depends on the substrate-induced electromagnetic screening effect and the hybridization between graphene and the substrate.[45] As a result of the relatively stronger suppression for Gr/Pt(111) (in particular, the intensity of 2D band is a factor of $\sim$100 weaker in comparison to Gr/Cu(111)),[45] the oxygen stretching band can be ob-

served at ≈ 1554 cm^{-1}.[46] Lastly, we note that the different spectral backgrounds observed in Gr/Pt(111) and Gr/Cu(111) are associated with photoluminescence induced by the metallic substrates.[44]

Quantitative analysis is carried out by subtracting a linear background from the spectra, and the Raman characteristic bands are fitted with a Lorentzian lineshape to extract their peak intensities. Two intensity ratios, I_D/I_G and I_D/I_{2D},[40–43] are used as indicators to evaluate the relative disorder concentrations of different samples (table S1 and S2). In particular, I_D/I_{2D} is used here because the intensity of the 2D band (originating from the intervalley double resonance process of graphene lattice) decreases monotonically as a function of the concentration of disorder,[43] and only depends weakly on the doping (n- or p-type);[47] in contrast to the intensity of the G band, which exhibits a two-stage transition as a function of disorder: (1) in the low-disorder regime, it remains basically constant with increasing disorder; (2) in the high-disorder regime, it decreases drastically and smears out eventually.[40] As can be seen in Table S1 and Table S2, generally, both ratios increase with the mass of implanted gas, i.e. $Ar > Ne > He$, regardless of the underlying substrate.

Table S1: The D versus G band intensity ratio (I_D/I_G) of pristine and noble gas (He, Ne and Ar) implanted graphene, on two types of substrates, including Pt(111) and Cu(111).

Gr/substrate	I_D/I_G			
	pristine	He	Ne	Ar
Gr/Pt(111)	0	≈0	0.97	1.86
Gr/Cu(111)	0	≈0	1.5	3.47

Table S2: The D versus 2D (G′) band intensity ratios (I_D/I_{2D} or $I_D/I_{G'}$) of pristine and noble gas (He, Ne and Ar) implanted graphene, on two types of substrates, including Pt(111) and Cu(111).

Gr/substrate	I_D/I_{2D}			
	pristine	He	Ne	Ar
Gr/Pt(111)	0	≈0	3.26	6.34
Gr/Cu(111)	0	≈0	3.15	3.35

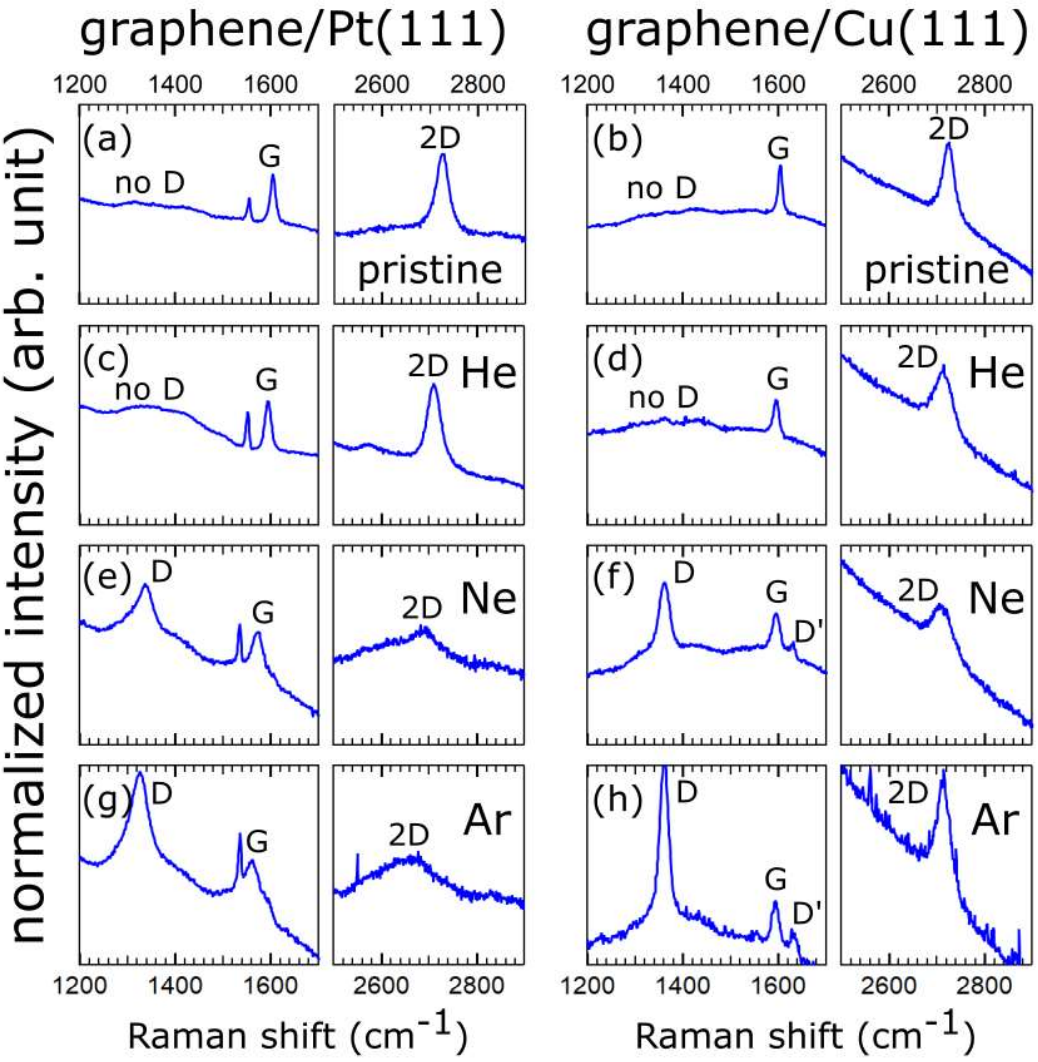

Figure S6: Representative Raman spectra (laser wavelength = 532 nm) of graphene on Pt(111) (a, c, e, g) and on Cu(111) (b, d, f, h), where both pristine graphene (a, b) and noble gas implanted graphene (c, d, e, f, g, h) are presented. The following noble gases are implanted: (c, d) He; (e, f) Ne; (g, h) Ar. The spectra are normalized by the intensity of G band. The normalized intensity of 2D (G′) band (right panel of each spectrum) is multiplied by a factor of 2, for visualization purpose.

Effect of fluence

Figure S7 shows STM topographies from two Ne-implanted samples: one corresponding to the data presented in the main text (implanted with a fluence of 1×10^{15} ions per cm^2) and an additional sample implanted with a higher fluence (3×10^{15} ions per cm^2). As expected, the sample with higher fluence appears to exhibit a higher density of bubbles. We note however that further studies are required to quantitatively assess the relation between fluence and bubble properties such density and size.

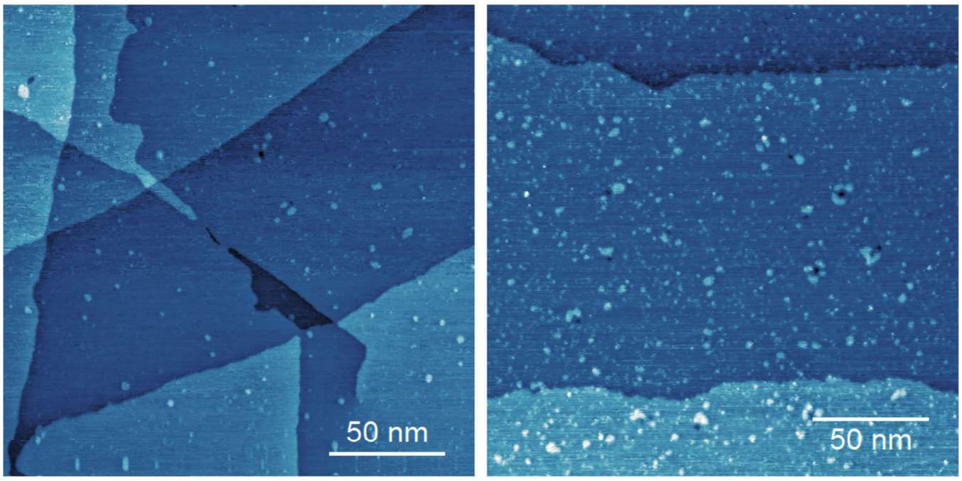

Figure S7: (Left) STM topography of Ne bubbles in graphene on Pt(111) with a fluence of 1×10^{15} ions per cm^2. (Right) STM topography of Ne bubbles in graphene on Pt(111) with a fluence of 3×10^{15} ions per cm^2.

Graphene domains

Figure S8 shows the low-energy electron diffraction (LEED) patterns for further characterization of the domain structure of the graphene/Pt(111) system. In (a), the diffraction patterns (corresponding to the graphene lattice obtained at 143 eV) for a pristine sample show two prominent domains surrounded by discontinuous rings symmetrical to the dominant spots (indicated along the red arc). These ring-like features can be attributed to the presence of a distribution of rotational domains, such as those observed for similar systems.[48,49] The diffraction spots corresponding to the Pt(111) surface lattice (indicated by yellow arrows)

are observed closer to the center of the pattern compared to the graphene spots (which are misaligned with respect to the Pt lattice) owing to the larger real space surface lattice parameter of platinum. These spots are more visible in the inset (acquired at 205 eV) displaying the three-fold symmetric patterns from the Pt(111) thin film. From the spot intensity profile as shown in (b), acquired along the red arc in (a), we can see that there is a distribution of rotated domains, spread around two main orientations separated by 16.5°.

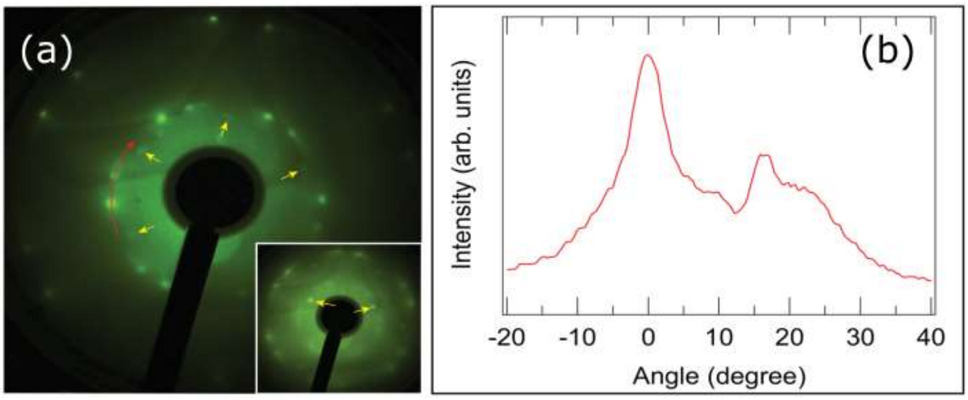

Figure S8: (a) LEED pattern obtained at 143 eV (inset at 205 eV) for pristine graphene/Pt(111) shows the characteristic hexagonal spots (yellow arrows) from Pt(111) and the diffraction spots from two main graphene domains, surrounded by the discontinuous ring like features (red arc). (b) Intensity profile along the red arc in (a).

Estimation of tensile strain

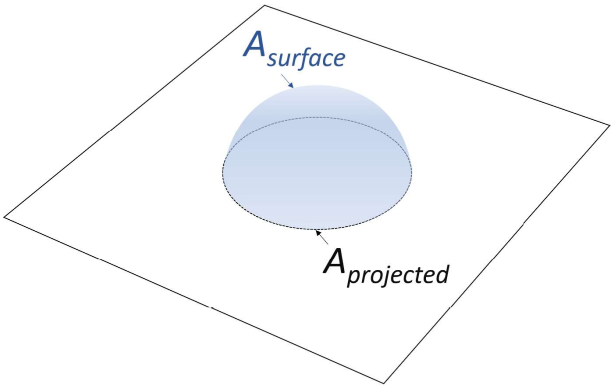

Figure S9: Illustration of how the surface area of a bubble ($A_{surface}$) and the projected area of its base ($A_{projected}$) are defined.

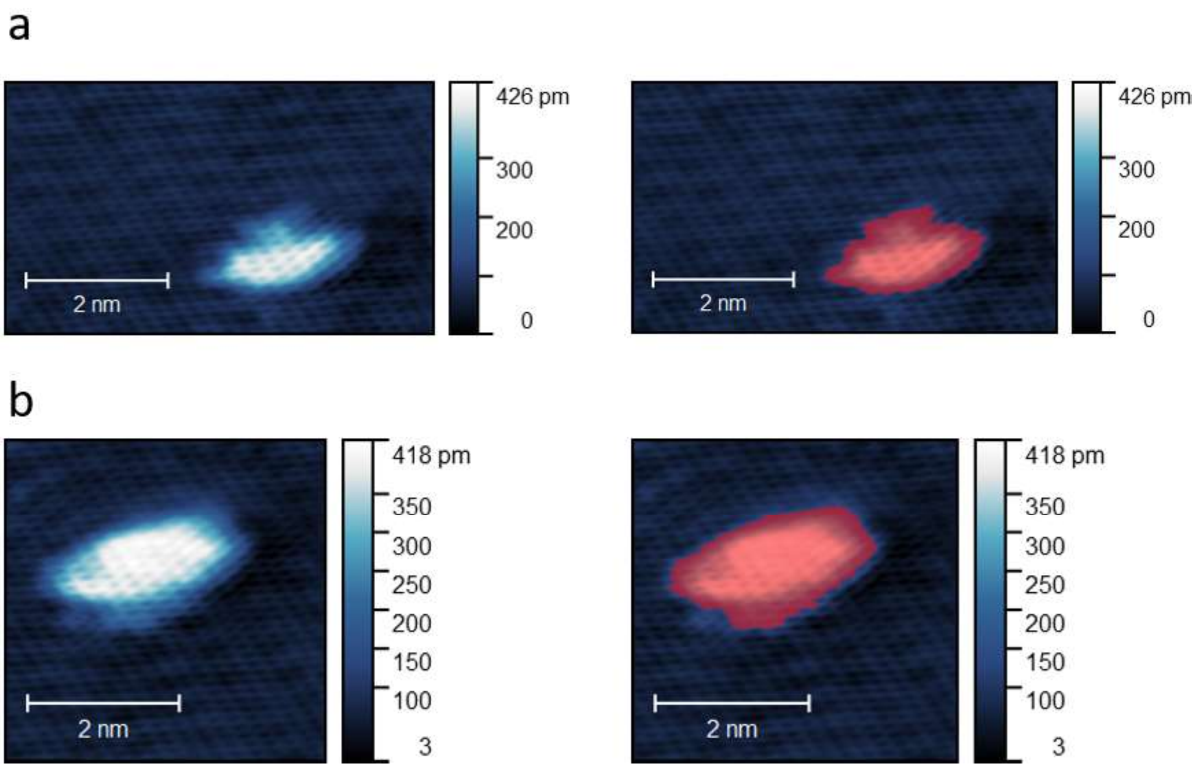

Figure S10: STM topographies of two He bubbles (a and b) in graphene on Pt(111). The panels on the right correspond to the same data as in the panels on the left, with the surface area selection (i.e. the region considered as a bubble) highlighted in red.

As mentioned in the main text, we estimated the tensile strain induced on graphene by the underlying trapped atoms from STM topographies of two bubbles, by determining the surface area of the graphene layer that wraps the 3-dimensional bubble ($A_{surface}$), as well as the (projected) area of the base of the bubble ($A_{projected}$) (Fig. S9). We selected high-quality STM data of two He bubbles exhibiting smooth, low-noise, atomic-resolution STM topography (Fig. S10). In order to obtain the area values, the raw STM data is process as follows: the micrograph is leveled by mean-plane subtraction, the rows are aligned, the minimum height is set to zero, and the topography is filtered (using the 2D FFT) to minimize noise spikes that would otherwise result in an overestimation of $A_{surface}$. The tensile strain of the bubble is estimated by selecting the area of the bubbles as shown in red in Fig. S10 and using the following equation:

$$\epsilon_A = \frac{\sqrt{A_{surface}} - \sqrt{A_{projected}}}{\sqrt{A_{projected}}}. \tag{7}$$

Using this approach, we obtain values of tensile strain of $\epsilon_A \approx 12$ % for the bubble in Fig. S10a and $\epsilon_A \approx 9$ % for the bubble in Fig. S10b.

Density estimation

Based on our MD simulations, we estimated the density of the trapped atoms inside the bubbles, taking two extremes, for large and small bubbles, *i.e.* with 800 and 10 atoms, respectively. The density was estimated as the ratio of the total mass of trapped atoms to the volume occupied by the elements. The occupied volume was calculated by performing a Voronoi tessellation over the coordinates of all the atoms and determining the volume of the Voronoi cells corresponding to the noble gas atoms. The estimated density values are summarized in Table S3. These values agree well with experimentally determined densities of solid He,[58] Ne[59] and Ar[60] at such high pressure (few to tens of GPa). A more detailed comparison is however challenging, given the different dimensionality of the systems (nan-

ocluster versus bulk) and the difficulty in unambiguously defining the volume occupied by the trapped atoms.

We also calculated the fraction of volume occupied by the trapped elements inside the bubbles. The fraction is estimated as the ratio of the volume occupied by the trapped atoms (as used for the density calculations) to the corresponding bubble volume. The bubble volume is determined using numerical integration of the graphene profile to yield the volume between the graphene surface and the plane defined by the graphene layer away from the bubble. The calculated volume fraction values are also summarized in Table S3.

Table S3: Density and volume fraction of the trapped atoms, for large and small bubbles, *i.e.* with 800 and 10 atoms, respectively, estimated based on our MD simulations

Element (number of atoms)	Density (kg/m^3)	Volume fraction
He (10)	908	0.81
Ne (10)	2476	0.64
Ar (10)	3328	0.40
He (800)	756	0.89
Ne (800)	2574	0.84
Ar (800)	2908	0.84

Adsorption parameters for He, Ne and Ar on Pt(111) and Cu(111)

We performed density functional theory calculations to determine the adsorption energy and distance of He, Ar and Ne over Pt(111) and Cu(111). Figure S11 shows the computed supercells and corresponding adsorption sites, as well as the electron density isosurfaces. There are four important high-symmetry adsorption sites: top, bridge, hcp and fcc. The obtained values for adsorption energy (E_a) and adsorption distance (d) are listed in Table S4.

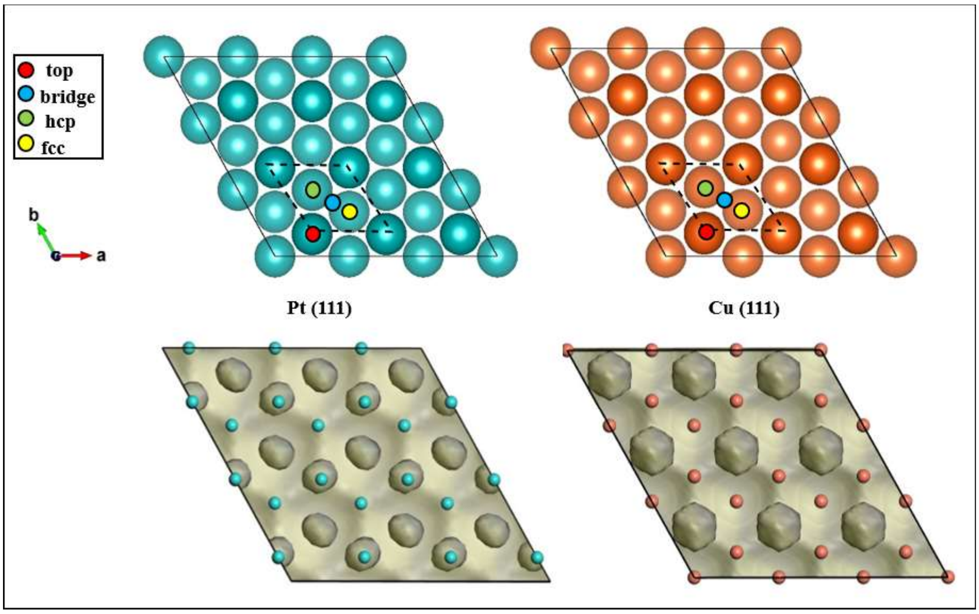

Figure S11: The top panels show the high-symmetry adsorption sites for the Pt(111) and Cu(111) planes when X gas (X = He, Ne, and Ar) is adsorbed. Bottom panels show the electron density isosurfaces for both Pt(111) and Cu(111) planes (isovalue taken to be 0.2 $e/Å^3$). The Pt and Cu atoms are shown in green and orange, respectively.

Table S4: Results fro the DFT calculations: adsorption energy (E_a) in eV, and adsorption distance (d) in Å, for X gas adsorbed (X= He, Ne, and Ar) on the high-symmetry sites in Pt(111) and Cu(111).

substrate	site	Ar		Ne		He	
		E_a	d	E_a	d	E_a	d
Cu(111)	top	-0.319	3.30	-0.088	3.306	-0.077	3.305
	bridge	-0.287	5.13	-0.111	4.992	-0.099	5.062
	hcp	-0.319	3.38	-0.097	3.178	-0.076	3.169
	fcc	-0.319	3.45	-0.097	3.131	-0.076	3.245
Pt(111)	top	-0.461	3.588	-0.157	3.606	-0.131	2.129
	bridge	-0.458	3.729	-0.136	3.375	-0.111	3.430
	hcp	-0.484	3.458	-0.145	3.477	-0.113	3.562
	fcc	-0.480	3.460	-0.137	3.449	-0.110	3.485
